# How do tasks impact the reliability of fMRI functional connectivity?

**DOI:** 10.1002/hbm.26535

**Published:** 2024-02-13

**Authors:** Shefali Rai, Kirk Graff, Ryann Tansey, Signe Bray

**Affiliations:** ^1^ Child and Adolescent Imaging Research Program University of Calgary Calgary Alberta Canada; ^2^ Alberta Children's Hospital Research Institute University of Calgary Calgary Alberta Canada; ^3^ Hotchkiss Brain Institute University of Calgary Calgary Alberta Canada; ^4^ Department of Neuroscience University of Calgary Calgary Alberta Canada; ^5^ Department of Radiology University of Calgary Calgary Alberta Canada

**Keywords:** BOLD signal, fMRI, functional connectivity, precision, test–retest reliability

## Abstract

While there is growing interest in the use of functional magnetic resonance imaging‐functional connectivity (fMRI‐FC) for biomarker research, low measurement reliability of conventional acquisitions may limit applications. Factors known to impact FC reliability include scan length, head motion, signal properties, such as temporal signal‐to‐noise ratio (tSNR), and the acquisition state or task. As tasks impact signal in a region‐wise fashion, they likely impact FC reliability differently across the brain, making task an important decision in study design. Here, we use the densely sampled Midnight Scan Club (MSC) dataset, comprising 5 h of rest and 6 h of task fMRI data in 10 healthy adults, to investigate regional effects of tasks on FC reliability. We further considered how BOLD signal properties contributing to tSNR, that is, temporal mean signal (tMean) and temporal standard deviation (tSD), vary across the brain, associate with FC reliability, and are modulated by tasks. We found that, relative to rest, tasks enhanced FC reliability and increased tSD for specific task‐engaged regions. However, FC signal variability and reliability is broadly dampened during tasks outside task‐engaged regions. From our analyses, we observed signal variability was the strongest driver of FC reliability. Overall, our findings suggest that the choice of task can have an important impact on reliability and should be considered in relation to maximizing reliability in networks of interest as part of study design.

## Graphical abstract

We used the densely sampled Midnight Scan Club dataset to investigate how tasks impact functional magnetic resonance imaging (fMRI)‐functional connectivity (FC) reliability, considering regional task effects and task‐driven changes in signal properties, such as temporal standard deviation (tSD). Relative to rest, we found that tasks only enhanced fMRI‐FC reliability and tSD for specific task‐engaged regions.

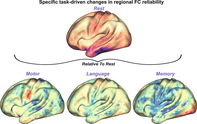

## 1 INTRODUCTION

Functional connectivity (FC), typically calculated as the correlation between functional magnetic resonance imaging (fMRI) time courses from different parts of the brain (Biswal et al., 1995; Power et al., 2011; Yeo et al., 2014), reflects both relatively stable and dynamic time‐varying characteristics (Gratton et al., 2018). As fMRI‐FC can capture stable interindividual variation in functional brain organization, there is tremendous interest in both describing brain network alterations associated with psychiatric and neurological disorders (Grimm et al., 2021; Lam et al., 2022; Runia et al., 2022) and developing biomarkers for diagnosis and treatment (Sui et al., 2020; Uddin et al., 2017; Woo et al., 2017). Despite excitement for the clinical potential of fMRI‐FC, challenges with replication (Dinga et al., 2019; King et al., 2019) and reproducibility (Marek et al., 2022; Poldrack et al., 2017) present an obstacle to clinical translation. Measurement reliability of conventional acquisitions has been noted as a challenge, as this property places an upper bound on effect sizes, impacting study power (Marek et al., 2022; Noble et al., 2021) and potentially impacting reproducibility (Szucs & Ioannidis, 2020). Further, low reliability limits clinical applications where reliable within‐person measurements would be necessary for diagnosis, treatment decisions or characterization of treatment effects (Gratton et al., 2020, 2022). A better understanding of the factors that impact FC reliability can therefore help to improve experimental designs in this field.

Test–retest reliability generally refers to the extent to which a measurement produces a similar value when repeated under similar conditions, while validity refers to the capacity of a measurement to assess the construct of interest. BOLD fMRI is an inherently noisy measure with several factors known to influence both reliability and validity of measurements. Further, head motion (Power et al., 2012) leads to systematic alterations to the functional connectome that reduce validity, but may artificially increase reliability (Noble et al., 2019; Parkes et al., 2018). FC is often calculated using correlations, which become more accurate with increasing sample size (Schönbrodt & Perugini, 2013), and it has been shown that FC reliability increases asymptotically with an increasing number of timepoints acquired (Gordon et al., 2017; Laumann et al., 2015; Noble et al., 2019; Noble, Scheinost, et al., 2017; Shah et al., 2016). Another factor known to influence FC reliability is task, with work showing differences in FC reliability when comparing task to resting‐state acquisitions at the whole connectome level (Patriat et al., 2013; Wang, Ren, et al., 2017) and in specific subnetworks (O'Connor et al., 2017; Zou et al., 2015), though how reliability changes relate to region‐wise task effects has, to our knowledge, not been comprehensively explored.

The impact of task on FC reliability is important to consider, as time in the scanner is limited and task is a factor the experimenter can control. While a typical fMRI scan length consists of acquiring 10 min of data per person, longer acquisitions lengths, of greater than 30 min, asymptotically increase reliability (Laumann et al., 2015). However, long resting‐state scans are vulnerable to head motion (Satterthwaite et al., 2012; Vanderwal et al., 2015) and uncontrolled variations in drowsiness (Tagliazucchi & Laufs, 2014). Increasingly, tasks are used as an alternative to resting‐state acquisitions to increase participant engagement and reduce head motion (D. J. Greene et al., 2018) and elicit states of interest (Finn, 2021). Many studies collect fMRI data across multiple rest and task acquisitions and concatenate the data (J. Chen et al., 2022; Gao et al., 2019) to increase data quantity toward improved reliability (Elliott et al., 2019, 2020; Herting et al., 2018; Tetereva et al., 2022). This work underscores the importance of considering how tasks can impact fMRI‐FC measurements.

FC reliability varies across the brain (Noble, Spann, et al., 2017; Yeo et al., 2011) and is particularly low in regions with high signal dropout, due to susceptibility artifacts, including orbitofrontal cortex and anterior temporal regions (Yeo et al., 2011). Temporal signal‐to‐noise ratio (tSNR), the ratio of signal mean to temporal standard deviation (tSD), has been linked with variations in FC reliability across the brain (Yeo et al., 2011), though this relationship has been noted to be nonlinear (Mueller et al., 2015). Tasks are associated with both increases and decreases in BOLD signal variation (Ito et al., 2020), which is one component of tSNR. Here, we consider whether changes in BOLD signal variation are related to task‐driven changes in FC reliability.

In the present study, we leverage the densely sampled Midnight Scan Club (MSC) dataset (Gordon et al., 2017) to investigate how tasks affect regional FC reliability across the cortical surface and within functional networks. This dataset provides fairly long time courses of both task and resting‐state data, enabling relatively accurate estimates of test–retest reliability. Prior work has emphasized the relationship between acquisition time and reliability (Gordon et al., 2017; Gratton et al., 2018; Laumann et al., 2015). Given that the MSC dataset lies within the higher range of the asymptotic reliability curve, our analyses between task and FC reliability were less influenced by scan length. We hypothesized that task states designed to engage specific brain regions may enhance reliability of FC within those regions. To further elucidate the factors contributing to FC reliability, we considered how task effects and BOLD signal properties, specifically mean signal and tSD (Table 1 defines measures used in this study), associate with reliability. Together our findings suggest that tasks may impact FC reliability in a manner highly specific to region and context and can help to understand the advantages and disadvantages of using tasks for FC studies.

**TABLE 1:** Glossary of fMRI measures.

Measure name	Other acronym(s)	Definition
Functional magnetic resonance imaging‐functional connectivity	fMRI‐FC, FC	Statistical dependence, typically correlation, between fMRI time courses from distinct brain regions during rest or task performance
Test–retest correlation	FC‐TRC, FC reliability	Spatial Pearson correlation between split half (sessions 1–5 and sessions 6–10) functional connectivity connectomes on a vertex level
Relative test–retest correlation	∆ FC‐TRC, relative FC‐TRC, delta FC‐TRC	Subtraction of test–retest correlation between each task state and rest
Intraclass correlation	ICC, ICC (2,1), FC reliability	Division between interindividual variation by the total variation using split half (sessions 1–5 and sessions 6–10) functional connectivity connectomes on a parcel level
Temporal mean signal	tMean	Averaged temporal mean signal of BOLD time courses across all sessions
Relative temporal mean signal	∆ tMean, relative tMean, delta tMean	Subtraction of temporal mean signal between each task state and rest
Temporal signal‐to‐noise ratio	tSNR	Ratio of temporal signal mean to temporal standard deviation
Temporal standard deviation	tSD	Averaged temporal standard deviation of BOLD time courses across all sessions
Relative temporal standard deviation	∆ tSD, relative tSD, delta tSD	Subtraction of temporal standard deviation between each task state and rest
Parameter estimates	Task effects, PEs	Beta estimates of task condition regressors obtained from group‐level general linear models

## 2 METHODS

### 2.1 Participants

For this study, we used the open‐source MSC dataset. The current analyses utilized data from 9 healthy adults (M = 5, F = 4) aged 24 to 34 years of age (mean = 29.3; SD = 3.5) that were recruited from within the Washington University community. Two of these participants are authors of the original MSC study by Gordon and colleagues (Gordon et al., 2017). For the original MSC study, a total of 10 adults (M = 5, F = 5) participated in 12 separate sessions. During the first two sessions, the study collected four T1‐weighted, four T2‐weighted images, four MR angiograms, and eight MR venograms. During the last 10 sessions, the study collected fMRI data.

### 2.2 Data collection

Data were collected using a Siemens TRIO 3 T MRI scanner over a period of 12 sessions on separate days, each commencing at midnight. fMRI scan data from task and resting‐state scans were analyzed and T1‐weighted and T2‐weighted images were used to preprocess fMRI scans. Four T1‐weighted scans (0.8 mm isotropic, TR = 2400 ms, TE = 3.74 ms, T1 = 1000 ms, FA = 8°, 224 sagittal slices) and four T2‐weighted scans (0.8 mm isotropic, TR = 3200 ms, TE = 479 ms, 224 sagittal slices) were acquired per participant. Details on the MRA and MRV scans are provided in the original MSC study (Gordon et al., 2017).

Functional images were acquired using a gradient‐echo EPI BOLD sequence (TR = 2200 ms, TE = 27 ms, FA = 90°, voxel size = 4 × 4 × 4 mm^3^, 36 axial slices), with one gradient field map sequence collected in each session with the same prescription as the functional scans. For each participant, a total of 300 min of rest fMRI and 350 min of task fMRI scans were collected over 10 subsequent days. An eye‐tracker camera was used to assess participant wakefulness. In line with previous studies (Gordon et al., 2017; Gratton et al., 2018), one participant, MSC08, was excluded from this study due to self‐reported sleep, prolonged eye closures, and a large amount of head motion.

### 2.3 fMRI rest and task details

#### 2.3.1 Rest

For resting‐state scans, participants were asked to fixate on a white crosshair against a black background. These data were acquired in one continuous 30‐minute run per session for a total of 300 min across 10 days.

#### 2.3.2 Motor task

The motor task was adapted from the Human Connectome Project (Barch et al., 2013), where in each block participants were instructed to either close or relax their left or right hand, flex and relax their left or right foot, or wiggle their tongue. Each motor block began with a 2.2 s cue instructing the participant of which movement was required. Following this instruction, a fixation caret—using the (^) symbol—was placed in the center of the screen and each time the caret flickered, every 1.1 s, the participant executed the movement required. A total of 12 movements occurred per block and each task run comprised 2 blocks of each type of hand, food, or tongue movement, with 3 blocks of resting fixation for a total of 15.4 s. Two runs of the motor task were conducted per session for a combined total of 78 min across 10 days.

#### 2.3.3 Language task

The language task was adapted from Dubis et al. (2016) and consisted of two mixed block and event‐related tasks presented within the same run. The first task was the spatial coherence discrimination task, which asked participants if the presented dot patterns were organized randomly or concentrically. The second task was the verbal discrimination task, in which participants were asked if a presented word was a verb or noun. Each block started with a 2.2 s cue informing participants of the upcoming task to be performed and ended with a 2.2 s cue indicating the end of each block. Blocks, for both spatial coherence and verbal tasks, comprised 30 trials (half concentric and half nonconcentric, half noun and half verbal) and stimuli were shown for 0.5 s. Each run included two blocks of each task, lasting 14.2 min total, with a 44 s fixation period in between each task block. For this task, the finger used for each participant's decisions was counterbalanced within participants across sessions. Two runs of the language task were presented per session for a combined total of 142 min across 10 days.

#### 2.3.4 Memory task

The incidental encoding or memory task required participants to view 24 images of either faces, scenes, or words, repeated 3 times (e.g., face‐first, face‐second, and face‐third repeat). Stimuli were shown for 1.7 s with a jittered interstimulus interval that ranged between 500 and 4900 ms. For the face runs, participants were asked if the presented face was female or male; on the scene runs, participants were asked if the presented scene was outdoor or indoor; and for the word runs, participants were asked if the presented word was abstract or concrete. Participants were asked to respond as soon as possible, regardless of the number of times each stimulus was shown. Participants made responses using a fiber‐optic response box. Again, as with the language task, the finger used for decisions was counterbalanced within participants across sessions. Three runs of the memory task were performed per session for a combined total of 131 min across 10 days.

### 2.4 Data and code availability

The MSC dataset was obtained from OpenNeuro (https://openneuro.org/datasets/ds000224/). Python, R, and MATLAB scripts used in this study are available at https://github.com/BrayNeuroimagingLab/BNL_open.

### 2.5 Preprocessing

Preprocessing was done using a custom pipeline using Nipype (Gorgolewski et al., 2011) version 1.5.0 integrating functions from FSL version 6.0.0 (Smith et al., 2004), ANTs version 2.3.4 (Avants et al., 2011), and AFNI version 21.1.16 (Cox, 1996). For both the four T1‐weighted images and four T2‐weighted images preprocessing, FSL *FLIRT* (Greve & Fischl, 2009; Jenkinson et al., 2002; Jenkinson & Smith, 2001) was used to co‐register the images from the same individual to each other, and *fslmaths* was used to create averaged T1‐ and T2‐weighted images. ANTs *n4BiasFieldCorrection* was used to correct for intensity inhomogeneities, ANTs *BrainExtraction* to remove non‐brain tissues and the skull from images, ANTs *Registration* to warp each participant's brain to the adult MNI 152 nonlinear atlas, ANTs *Atropos* to generate tissue segmentations, and ANTs *ApplyTransforms* to warp segmentations back into native space. Then, *fslmaths* was used to create a mask of the white matter voxels, and AFNI's *3dmasktool* to erode tissue segments.

EPI preprocessing used FSL *slicetimer* for slice time correction, FSL *fugue* for field map distortion correction, FSL *MCFLIRT* (Jenkinson et al., 2002) for rigid body realignment, FSL *BET* for skull stripping, and FSL *FLIRT* boundary‐based registration to register the first session's EPI image to the averaged T1‐weighted image via the averaged T2‐weighted image. This atlas transformation was modeled from the procedure in Gratton et al. (2018), where the authors registered the mean intensity image from an EPI image to Talairach atlas space via the averaged high‐resolution T1‐ and T2‐weighted images. EPI images, from sessions 2 to 10, underwent the same steps using FSL BET and linear registration of each EPI image to the EPI reference image created from the first session.

We performed linear regression to remove the mean, linear, and quadratic trends from each voxel, band‐pass temporal filtering between 0.01 and 0.08 Hz. An additional filtering step was added after regression to remove high‐frequency motion (>0.1 Hz) in the phase‐encoding direction as described in Gordon et al. (2017) and Gratton et al. (2018). High motion frames, volumes above a framewise displacement (FD) threshold of 0.20 mm, were identified based on the approach from Power et al (Power et al., 2014). We then regressed out 24 head motion parameters, along with white matter, cerebrospinal fluid, and global signal regression (GSR). Though a controversial preprocessing step, we employed GSR because systematic pipeline comparisons suggest it is one of the most effective denoising tools available, when used in combination with other motion mitigation septs (Ciric et al., 2017; Graff et al., 2022; Parkes et al., 2018) and for consistency with the original MSC study (Gordon et al., 2017), which employed a whole brain regression step. Finally, ANTs *Registration* was used to warp the EPI images to MNI 152 nonlinear atlas space (Avants et al., 2011).

### 2.6 Cortical surface generation

Averaged T1‐weighted images were used to generate each participant's cortical surface using Freesurfer version 6.0's *recon‐all* pipeline (Dale et al., 1999). This pipeline registers each participant's brain to a template and performs the following steps: skull stripping, image registration, intensity normalization, and segmentation. Each participant's segmentation outputs were manually checked and edited as needed. Following Freesurfer, Ciftify (Glasser et al., 2013) version 2.3.3's *ciftify_recon_all* pipeline was used to convert Freesurfer outputs into grayordinate‐based analysis in the Connectivity Informatics Technology Initiative (CIFTI) format (Marcus et al., 2011). The grayordinate resolution for each participant is given in a low‐resolution mesh of ~32,000 vertices per hemisphere, parallel to the Human Connectome Project's standard space for fMRI analysis (Dickie et al., 2019;Marcus et al., 2011; Van Essen et al., 2012).

Using FSL *co‐register* and *fslmaths*, we created the volume averaged brain by combining T1‐weighted images from nine MSC participants. Following that, the FS *recon‐all* tool was used to create an FS averaged brain. After manually checking Freesurfer outputs, Ciftify was run on the FS averaged brain, which was then used to project group level measures, such as FC reliability and signal properties across the brain.

### 2.7 CIFTI fMRI data generation

Preprocessed fMRI time courses and task residuals were mapped to surfaces using Ciftify's *ciftify_subject_fmri* pipeline, which uses the ribbon‐constrained sampling procedure through the Connectome Workbench version 1.5.0's command line utilities (Marcus et al., 2011; Van Essen et al., 2012). This pipeline yielded surface mapped time courses that were spatially smoothed with a geodesic Gaussian kernel of σ = 4 mm, as recommended by the guidelines in Coalson et al. (2018) and excluded non‐gray matter tissue. Following surface mapping, identified high motion volumes with an FD > 0.20 mm were censored for each MSC participant and task (Supplemental Table 1) and used for subsequent analyses, executed in MATLAB version 9.11.0 (R2021b) (The MathWorks Inc., 2022a).

### 2.8 BOLD signal measures

We used censored and volume matched CIFTI time courses to calculate the following measures of BOLD signal properties across rest and all three tasks: temporal mean signal (tMean), tSD, and tSNR. Calculations were performed on a vertex‐wise level. Once calculations for all three measures were obtained for each vertex, we averaged across vertices for the parcel‐wise level. Using Connectome Workbench's *cifti‐parcellate* command line tool, we parcellated each participant's concatenated time courses using the 1000 parcel Schaeffer atlas based on the Yeo 17 network parcellation (Schaefer et al., 2018; Yeo et al., 2011). Across the surface brain maps, we depict vertex‐wise signal calculations and for statistical analysis, we refer to the parcel‐wise calculations to decrease computational load and further increase the tSNR.

We calculated tMean as the average values of the BOLD signal time courses concatenated across all 10 sessions for each participant. Similarly, we calculated tSD of the BOLD signal by averaging the standard deviation of the time courses across all 10 sessions for each participant. To obtain tSNR values, we averaged the BOLD signal time courses (tMean) and divided it by the standard deviation of the signal over time (tSD), across each scan run before averaging across all 10 sessions for a participant (Yeo et al., 2011). All metrics were calculated on both a vertex‐wise and parcel‐wise level. We calculated the average of each measure across sessions for each participant, and then averaged across participants within each parcel for comparison with FC‐TRC. Next, we computed ∆ tMean, by subtracting tMean at rest from tMean during each task. Similarly, ∆ tSD was calculated by subtracting tSD at rest from each task.

### 2.9 Connectome generation

We generated vertex‐wise connectomes for each participant and task separately, concatenating across days. For the resting‐state only analysis, we used all volumes retained after censoring. For the remaining analyses—comparing between rest and task states—we matched the length of all other tasks to the shortest motor task across sessions for all participants emphasized in red in Supplemental Table 2. Following concatenation across runs and days, vertex‐wise functional connectomes were created by calculating the Pearson correlation between the fully preprocessed and mean centered time courses, separately for rest and task scans. This resulted in a vertex‐wise 91,282 × 91,282 FC matrix for each participant. We also created parcellated FC connectomes which were used for group‐ and individual‐level network assignment. We then calculated the Pearson correlation of the parcellated time courses to produce a parcel‐wise 1000 × 1000 FC matrix for each participant.

### 2.10 Functional network assignment

Functional brain networks were mapped for the group‐averaged parcel‐wise FC matrix using similar approaches to those described in Gordon et al. (2017), Lancichinetti and Fortunato (2012), and Seitzman et al. (2018). Connectome diagonals were set to zero and edge density thresholds ranging from 2 to 5% were applied (Seitzman et al., 2019), setting the correlations below the threshold to zero and preserving values from other connections. Clustering community structures were acquired using Infomap, a network clustering algorithm (Rosvall et al., 2009). Infomap parameters consisted of using 1000 repetitions to increase accuracy and a two‐level partition of the networks. Consensus network communities were then found by combining all partitions produced by the Infomap algorithm with thresholds between 2 and 5% as outlined in Lancichinetti and Fortunato (2012). This procedure created a new consensus matrix by computing the probability of each vertex belonging to the same network across these density thresholds. For the group‐averaged network assignment, parcels belonging to ambiguous network outputs from Infomap, that is, networks over the expected 17‐network configuration, were reassigned to their neighboring parcel's network assignment. Parcel number 555 assigned by Infomap to network 18 and parcel number 908 assigned to network 19, were reassigned to networks 2 and 3, respectively.

Network definitions vary across individuals and datasets as previously shown (Gordon et al., 2017; Seitzman et al., 2019). Therefore, we manually assigned network names to each of the 17 networks obtained from the Infomap algorithm for the group‐averaged network assignment, using the network names described in the previous MSC study as a reference (Gordon et al., 2017). Networks were visualized for the group‐averaged brain on the inflated surfaces using Connectome Workbench (Marcus et al., 2011; Van Essen et al., 2012).

### 2.11 Assessing FC reliability for rest

To determine FC reliability during rest, we used two metrics of FC reliability: test–retest correlation (FC‐TRC), and intraclass correlation (ICC). Vertex‐wise functional connectomes were computed using split‐half sessions, that is, ~4000 time points after censoring the complete rest data. We divided the data using sessions 1–5 for the first half and sessions 6–10 for the last half. FC‐TRC was then calculated as the spatial Pearson correlation at each vertex for the two split‐half connectomes, producing test–retest vertex‐wise surface maps for every participant. Edge‐wise FC reliability was also computed, on a 1000 parcel level, across the dataset using ICC (ICC (2,1)). ICC (2,1) is defined as the absolute agreement between raters with random sources of error (G. Chen et al., 2018). ICC (2,1) was calculated by dividing the interindividual variation by the total variance using the ICC MATLAB toolbox (Salarian, 2022; The MathWorks Inc., 2022b). The two matrices used for ICC (2,1) consisted of the parcellated first half FC connectome and the parcellated last half FC connectome for each participant. ICC (2,1) is mathematically defined as:

$$\mathrm{ICC}\left(2,1\right)=p_{2}=\frac{\sigma_{\lambda }^{2}}{\sigma_{\pi }^{2}+\sigma_{\lambda }^{2}+\sigma_{\epsilon }^{2}}$$

In line with prior studies, ICC values are described using the following ranges: poor (0 < ICC ≤ 0.4), fair (0.4 < ICC ≤ 0.59), good (0.6 < ICC ≤ 0.74), and excellent (ICC ≥ 0.75) (Cicchetti & Sparrow, 1981).

### 2.12 FC reliability associations with BOLD signal properties

The relationship between FC‐TRC and BOLD signal properties were calculated across parcels for rest and all three tasks. For tSNR, we did not fit a linear model after visually gauging that a linear fit was inappropriate. We determined the relationship between tMean and tSD with FC‐TRC using linear regression across 1000 parcels. We repeated this analysis excluding low reliability networks (limbic A, B, and C) for tMean, tSD, and tSNR. A parallel analysis was conducted for task data with tMean and tSD on a parcel‐wise level. We also performed a linear regression analysis between tSNR and FC‐TRC on a network‐wise level, again excluding limbic networks A, B, and C, to extract standardized beta coefficients that could be compared across networks.

### 2.13 General linear model designs for task regression and task effects

FC is sometimes calculated from tasks after regressing out task effects, to focus on FC patterns that are not driven by shared effects of stimuli and to avoid inflated correlations (Cole et al., 2019). Although task regression is unlikely to capture all task‐evoked effects on FC (Fair et al., 2007), and may not enhance FC reliability (Cho et al., 2021), here, we conduct follow‐up analyses considering task state FC with regression of task effects. Following the approach from (Ito et al., 2020), we regressed the mean task‐evoked response from each condition's time courses using a finite impulse response (FIR) model, given its ability to reduce false positives for FC estimation (Cole et al., 2019; Ito et al., 2020). The FIR method's main advantage is that it does not make assumptions about the shape of the hemodynamic response function (Ollinger et al., 2001). For all first‐level and higher‐level statistical analyses, we used FSL's *FEAT* pipeline (Woolrich et al., 2001, 2004).

For each task condition, general linear model (GLM) analyses were conducted on preprocessed volume data, based on the procedure from Gratton et al. (2018). As suggested by Gordon et al. (2017), our study did not exclude incorrect responses across both language and memory tasks given the high level of accuracy across participants (Supplemental Table 3).

#### 2.13.1 Motor task

For each run of the motor task GLM, each experimental condition (left hand, right hand, left foot, right foot, and tongue) was modeled separately using an FIR basis function with seven time points at 2.2 s intervals in a 15.4 s window after the onset of the stimulus.

#### 2.13.2 Language task

For the mixed block language task GLM, each trial type and event were modeled separately. Both the verbal semantic and spatial coherence sustained signal were modeled using an FIR basis function with one time point in a 121 s window. Both the start and end cues in each task were modeled using an FIR basis function with one time point in a 2.2 s window. Finally, each trial type (50% coherent, 0% coherent [random], noun, verb) was modeled using an FIR basis function with eight time points at 1.5 s intervals in a 12 s window after the onset of the stimulus.

#### 2.13.3 Memory task

For the incidental encoding memory task GLM, each stimulus type (face, scenes, words) and repetition of each item (first, second, or third repeat) were modeled separately using an FIR basis function with eight time points at 1.5 s intervals in a 12 s window after the onset of the stimulus.

#### 2.13.4 Generating residualized connectomes

Residuals, from first‐level models, were then used to define task‐residualized connectomes, following the procedure described above for rest and task connectomes.

#### 2.13.5 Global effects of each task

To visualize regional effects of task on the BOLD response, each task's first‐level design created an averaged contrast using all time points for each condition (e.g., for the memory faces‐first repeat, each time point was given a weight of 1/8 [0.125] to average the condition into 1 contrast). These contrasts were then taken to the second‐level and combined across 10 sessions for each participant. We then conducted a third‐level, or group‐level analysis, by combining each separate contrast estimate obtained across all participants from the second‐level analysis (e.g., for the motor task, five contrast estimates, one for each motor task condition, were grouped together into one third‐level contrast for each participant). Outputs of group‐level analysis, that is, parameter estimates (PEs), or beta estimates, and z‐statistic images, for each contrast were projected onto the surface using Ciftify's *ciftify_vol_result* function. Next, we performed a similar linear regression analysis as mentioned above, between tSNR and PEs, on a network‐wise level, excluding limbic A, B, and C, to extract the standardized beta coefficients of each linear fit model across each network.

### 2.14 Task modulation of FC reliability

To better understand how tasks modulate FC‐TRC across the brain, we computed the difference between FC‐TRC for each of the three tasks relative to rest. Vertex‐wise functional connectomes were first calculated using split‐half sessions. We produced FC‐TRC vertex‐wise surface maps using the same procedure as rest data. Next, we compared relative FC‐TRC, or ∆ FC‐TRC, for each task to rest before and after task regression across the averaged brain.

### 2.15 Modeling variations in FC reliability

We used a linear mixed effect model to examine the relative importance of factors affecting FC reliability in each parcel, across subjects and task contexts. Using R version 4.2.2's (R Core Team, 2021) *lmer* function (Bates et al., 2015), we examined the influence of tMean, tSD, PEs, and modeled each participant as a random effect, calculated as:

$$\mathrm{FC}\;\text{reliability}\sim \text{tMean}+\mathrm{tSD}+\mathrm{PEs}+\left(1\;\right|\;\text{participant})$$

To facilitate comparison between predictors, we standardized all predictors in the model and set PEs (i.e., task effects) to zero for the rest condition. We visualized beta estimates for each factor in each parcel.

## 3 RESULTS

### 3.1 FC reliability during rest

**FIGURE 1:**
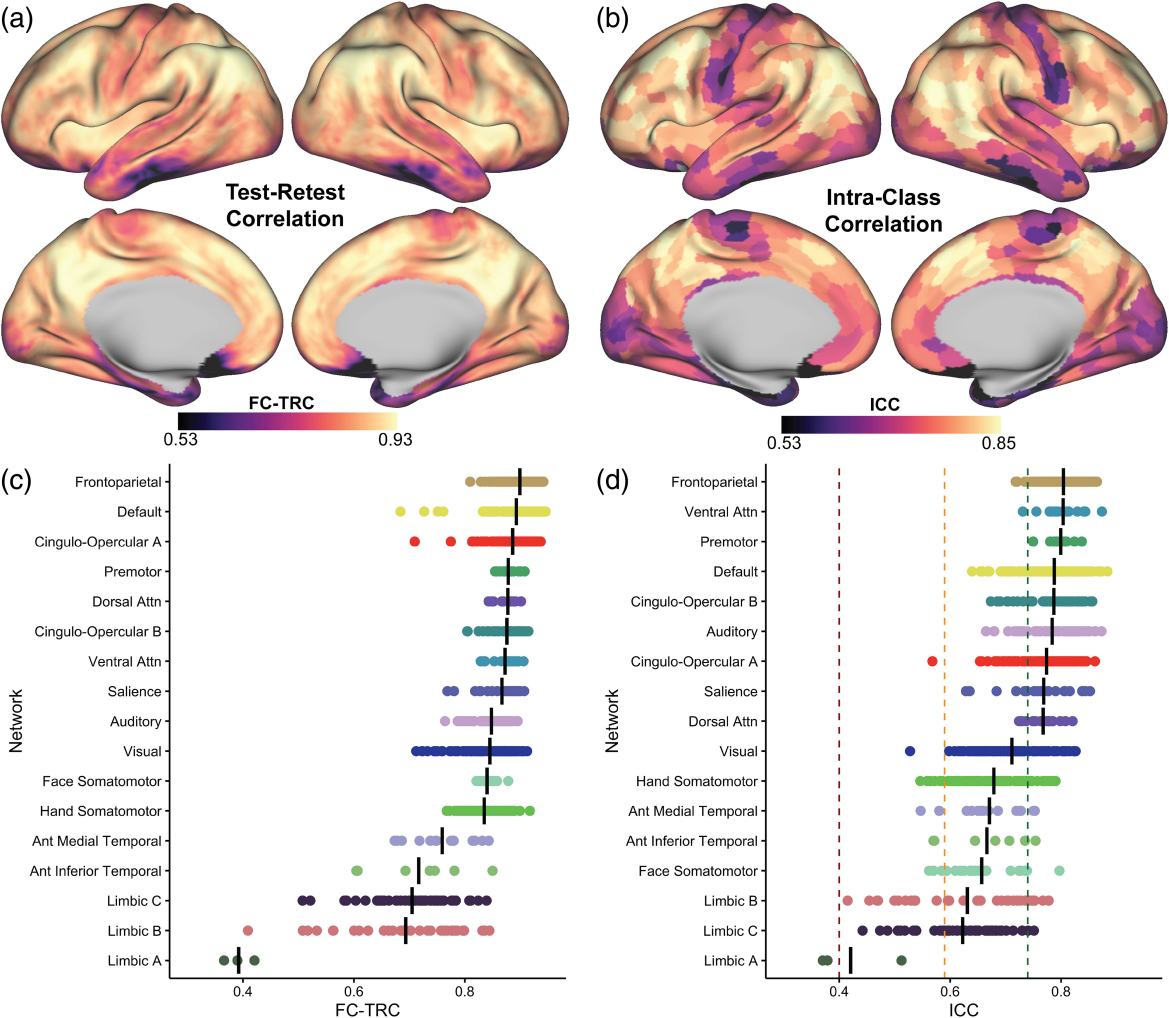
Greater test–retest correlation (FC‐TRC) and intraclass correlation (ICC) in higher order cognitive networks relative to sensory, motor, and limbic networks. (a) Variation in mean FC‐TRC is observed across the cortical surface. Brain regions such as precuneus, superior frontal lobe, and inferior parietal lobe exhibit higher FC‐TRC than regions of the motor cortex and temporal lobe. See Supplemental Figure 1 for FC‐TRC maps for individual participants. (b) Edge‐wise ICC depicted across the cortical surface on a parcel‐wise level paralleled the FC‐TRC cortical surface map. (c, d) Variation in reliability across networks using FC‐TRC (c) and ICC (d). Each dot represents a parcel belonging to 1 of the 17 functional networks (network topography outlined in Supplemental Figure 2). In (d), dotted lines demarcate the following ICC ranges: poor (0 < ICC ≤ 0.4), fair (0.4 < ICC ≤ 0.59), good (0.6 < ICC ≤ 0.74) and excellent (ICC ≥ 0.75).

The across participant mean of vertex‐wise FC‐TRC across the cortical surface are shown in Figure 1a. FC‐TRC maps for each of the nine MSC participants can be found in Supplemental Figure 1. Consistent with previous findings (Noble, Spann, et al., 2017; O'Connor et al., 2017), FC‐TRC varies substantially across the brain, and is highest in regions such as the precuneus, superior frontal lobe, and inferior parietal lobe, which are regions included in the default‐mode network (DMN) (Utevsky et al., 2014). Additionally, high FC‐TRC is visible in the middle frontal gyrus, a region of the frontoparietal network (FPN) (Marek & Dosenbach, 2018). In contrast, the motor cortex and temporal lobe regions, areas that are part of the somatomotor and limbic networks, respectively, have lower FC‐TRC. Figure 1c shows values averaged across regions assigned to 17 networks (networks shown in Supplemental Figure 2). Association networks had the highest mean FC‐TRC values, followed by sensory and motor networks, with limbic networks at the lowest mean FC‐TRC. Within‐network parcel‐wise ICC values depict a parallel trend across the cortical surface in 1B, with similar ordering of networks to the FC‐TRC plot in 1D. Association networks have “good” or “excellent” ICC values, while sensorimotor networks fall within the “fair” and “good” ICC range (orange and green dotted lines, respectively). Limbic networks (A, B, C), exhibited the lowest overall values, varying within the “poor” and “fair” ICC ranges (burgundy and orange dotted lines, respectively). The across‐participant standard deviation of FC‐TRC shows an opposite pattern (Supplemental Figure 3), where temporal lobe regions and areas of the motor cortex have higher variability across individuals, whereas regions of the DMN and FPN have lower variability. Regions with lower overall FC reliability also show the most variability across individuals.

### 3.2 Relationship between signal properties and FC reliability at rest

**FIGURE 2:**
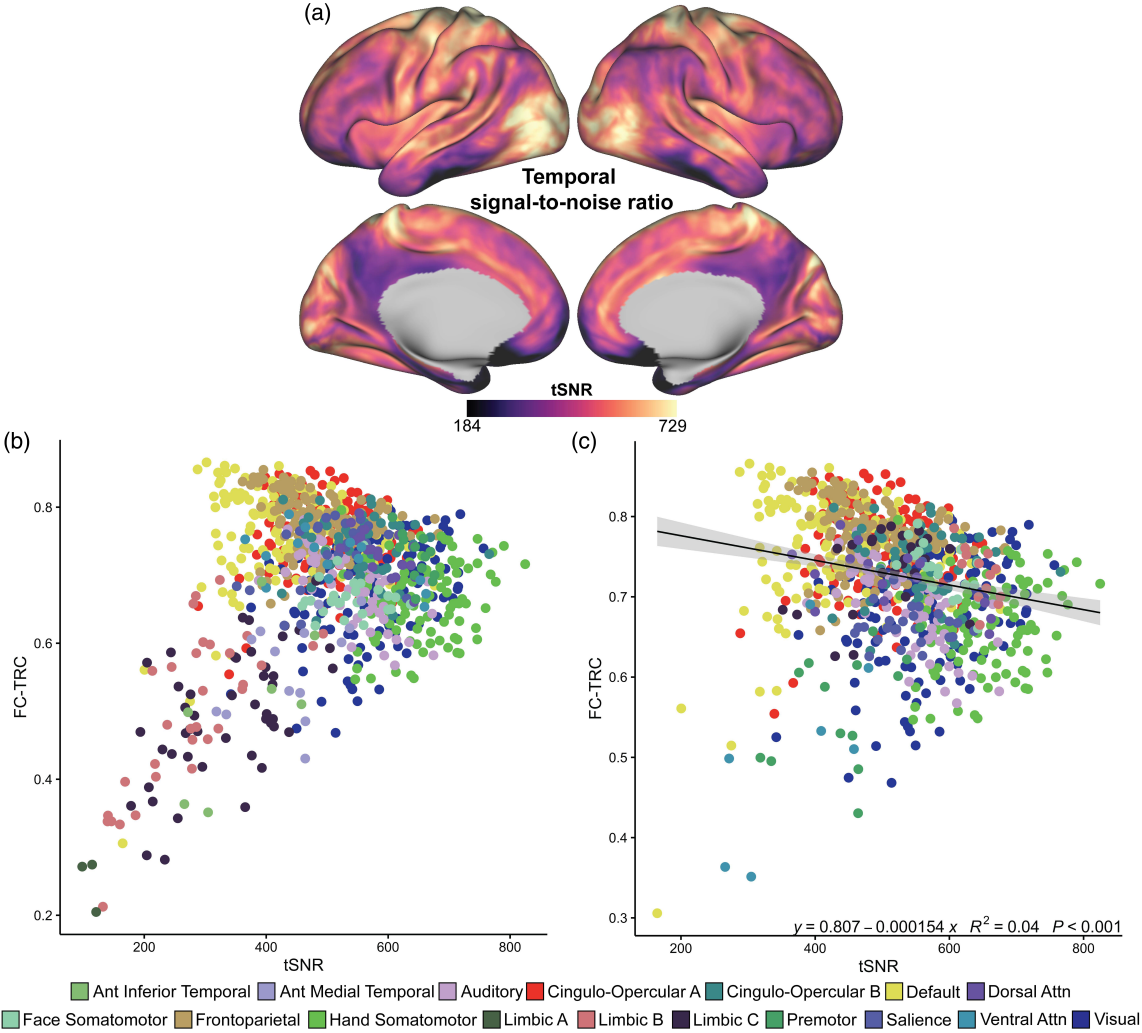
Temporal signal‐to‐noise ratio (tSNR) associates nonlinearly with functional connectivity (FC) reliability. (a) tSNR variations on a vertex‐wise level across the cortical surface during rest. (b) A nonlinear relationship is observed between tSNR and functional connectivity test–retest correlation (FC‐TRC), suggesting FC reliability is not solely dominated by tSNR. (c) Exclusion of low FC reliability networks (i.e., limbic A, B, and C networks) showed a significant and slightly negative relationship between tSNR and FC‐TRC.

We consider the relationship between FC reliability and regional signal properties at rest, in order to contextualize changes with tasks. First, we consider the relationship between tSNR and FC reliability. The distribution of tSNR across the cortical surface is shown in Figure 2a. In line with previous work (Mueller et al., 2015), the surface map depicted lower tSNR values for inferior temporal and inferior frontal regions that typically have larger signal drop‐out. We observe a nonlinear association between tSNR and FC‐TRC when all networks are considered (Figure 2b). After excluding low FC reliability networks (i.e., limbic A, B, and C networks), there was a small yet significant negative relationship (*β* = −1.54e^−4^, *R* ^2^ = .04, uncorrected *p* < .001) in Figure 2c. Further, we visualized the strength of this relationship within each network (Supplemental Figure 4a) and found highly variable relationships across networks. The overall negative trend between tSNR and FC‐TRC during rest is primarily dominated by attention networks, specifically dorsal and ventral attention network brain regions, and auditory network brain regions.

**FIGURE 3:**
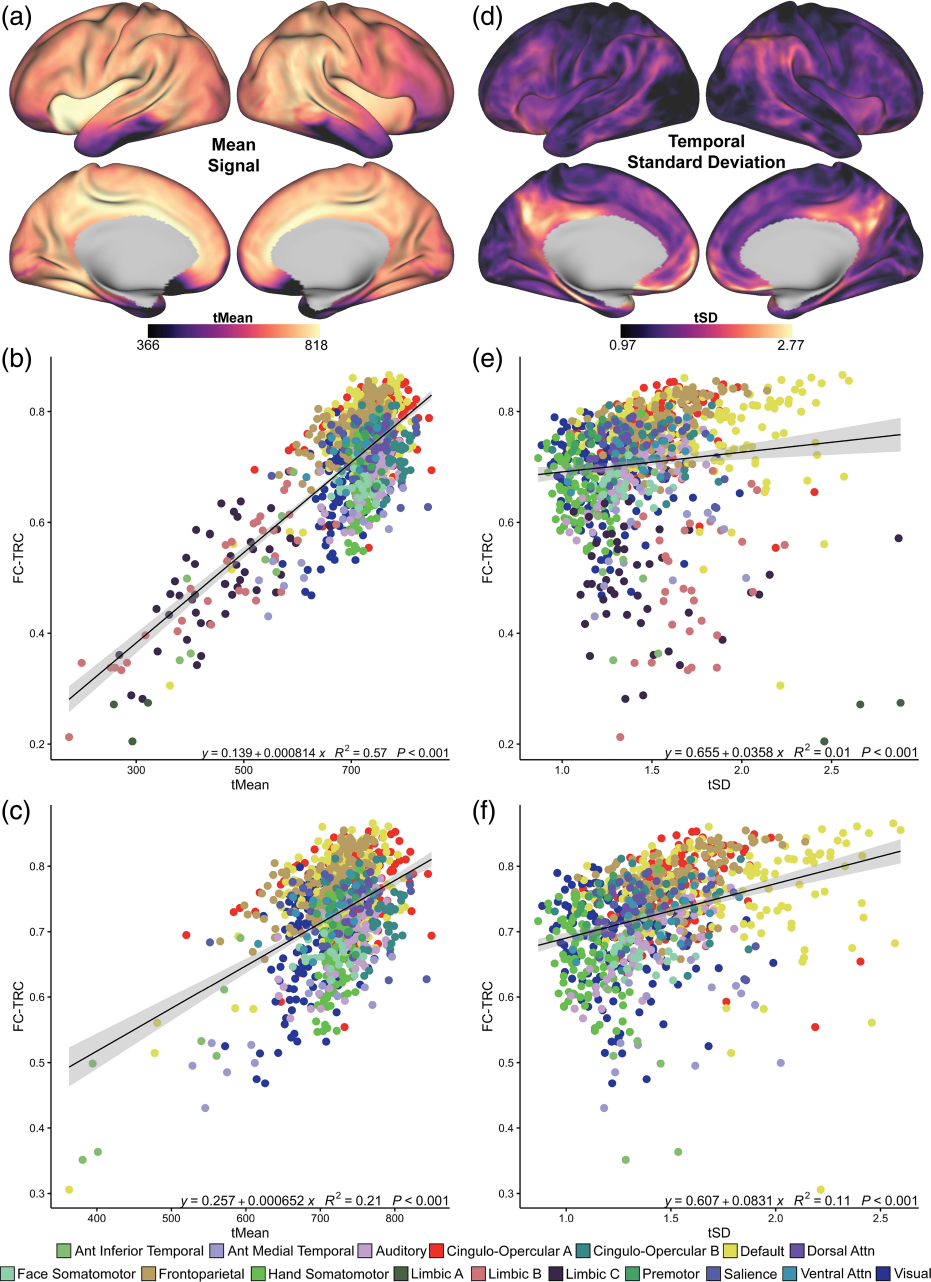
Regional variation in temporal mean signal (tMean) and temporal standard deviation (tSD) associate with functional connectivity test–retest correlation (FC‐TRC) during rest. (a, d) Group‐averaged tMean and tSD across the cortical surface. Parcel‐wise associations between FC‐TRC and tMean (b) and tSD (e) were fitted using linear regression. tMean associated positively with FC‐TRC across all parcels (b) and after excluding limbic networks (c). tSD had a small but significant relationship with FC‐TRC across all parcels (e), with a larger positive effect after excluding limbic networks (f).

Next, we separately assessed the two components of tSNR: tMean and tSD. Figure 3a,b show tMean positively relates to FC‐TRC (*β* = 8.14e^−4^, *R* ^2^ = .57, uncorrected *p* < .001), but this effect may be driven by limbic network regions (i.e., orbitofrontal and inferior temporal areas) with lower FC reliability and higher BOLD signal dropout (Glover, 2011; Olman et al., 2009). We therefore considered the relationship after excluding low FC reliability networks in Figure 3c. After thresholding, the relationship between FC‐TRC and tMean is attenuated but remains statistically significant (*β* = 6.52e^−4^, *R* ^2^ = .21, uncorrected *p* < .001). Figure 3d depicts tSD across the cortical surface and the association with FC‐TRC in 3E. tSD, in Figure 3e, did not show a clear relationship with FC‐TRC (*β* = 3.58e^−2^, *R* ^2^ = .01, uncorrected *p* < .001). However, after excluding low FC reliability networks in 3F, a significant positive relationship between tSD and FC‐TRC was found (*β* = 8.31e^−2^, *R* ^2^ = .11, uncorrected *p* < .001).

### 3.3 Task‐related changes in FC reliability

**FIGURE 4:**
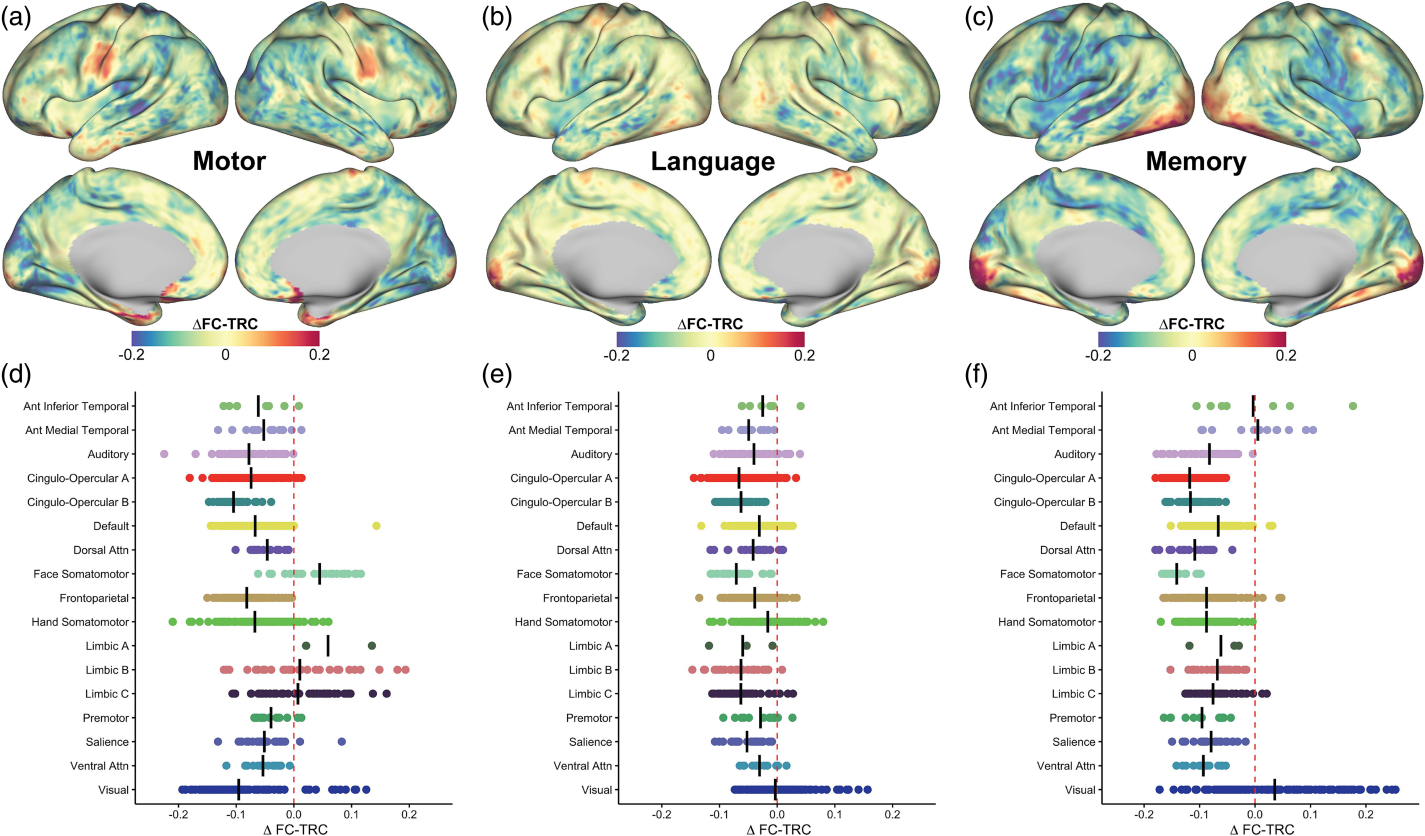
Tasks show regionally specific changes in relative functional connectivity (FC) reliability. Changes in FC‐test–retest correlation (TRC) for motor (a), language (b), and memory (c) tasks, relative to rest FC‐TRC. (d–f) Delta FC‐TRC, shown on a parcel‐wise level for each of the 17 networks, show broadly negative changes, with notable task‐influenced positive shifts in specific brain regions. For example, positive changes are observed in the face somatomotor network during the motor task (d), and the visual network during the memory task (f). Dotted lines demarcate the zero‐x‐axis line. Bolded lines indicate network mean delta FC‐TRC.

The relationships between tMean and tSD with FC‐TRC across all tasks were similar to rest (Supplemental Figure 5). When considering changes in FC‐TRC in tasks relative to rest, we found specific regional task‐related shifts in FC reliability for each task. Figure 4a–c shows vertex‐wise relative FC‐TRC, or ∆ FC‐TRC, between each task and rest, and Figure 4d–f depicts ∆ FC‐TRC in network parcels. Across tasks, FC‐TRC was generally attenuated with proportionally more parcels showing FC‐TRC decreases (more dots to the left relative to right of the zero dotted line in Figure 4d–f), with each task showing positive shifts in specific regions, rather than over whole networks. The motor task showed positive ∆ FC‐TRC for regions in the limbic (A, B, and C) and face somatomotor networks (Figure 4d). The language and memory tasks both showed prominent increases in FC‐TRC in visual region parcels. Across all tasks and networks, FC‐TRC decreased to a greater extent when task effects were regressed (Supplemental Figure 6). Given that regional FC reliability changes were task specific, we next considered how these variations may be related to task effects and modulations of regional signal properties.

### 3.4 Task modulation of regional activity and signal properties

**FIGURE 5:**
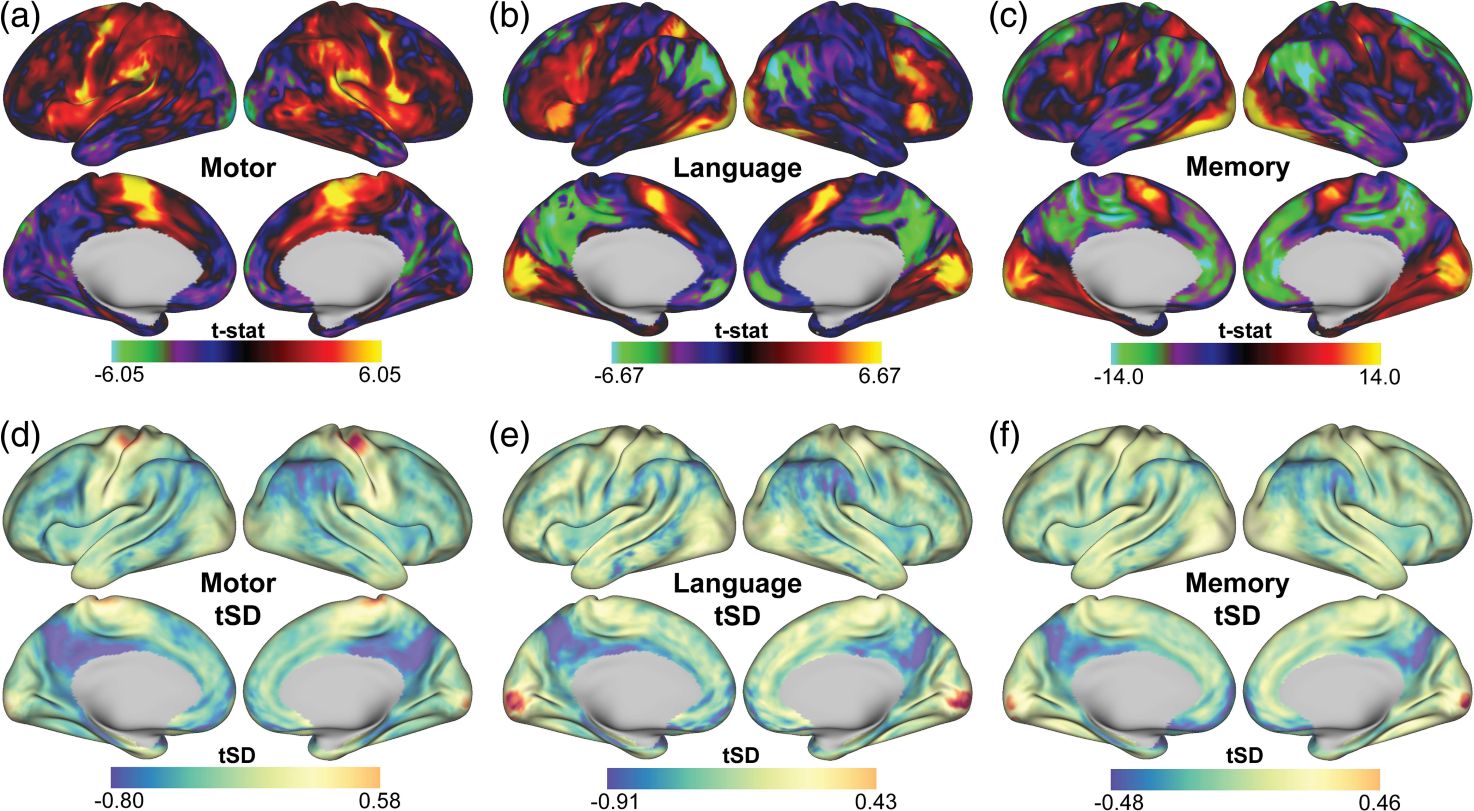
Regional task effects and changes in temporal standard deviation (tSD) across tasks. (a–c) Vertex‐wise task effect t‐contrast surface maps across all three tasks. The motor task had positive effects along the motor strip, the language task showed positive effects in visual regions and frontal/temporal regions with a left hemisphere bias, and memory task effects were most prominent in visual regions. (d–f) Vertex‐wise delta tSD was calculated as the difference between task and rest tSD. Relative to rest, task modulations of tSD partly reflected prominent task effects, for example, in motor (a, d), and visual regions (b, e and c, f).

Do tasks shift FC reliability specifically in the regions they engage, and does this occur via a change in signal properties? Figure 5 shows t‐contrasts of overall task effects across conditions in each of the three tasks. As expected, the motor task showed effects along the supplementary motor area and motor strip (Figure 5a), the language task in a left lateralized network that includes the posterior inferior frontal gyrus and Broca's area (Figure 5b), and for the memory task, task effects were apparent in regions known to associate with ventral and dorsal attention networks (posterior parietal cortex and dorsolateral prefrontal cortex) as well as visual regions (Figure 5c).

Although tMean associated with FC‐TRC across the brain at rest, positive changes in relative tMean during tasks were not significantly associated with increased FC‐TRC (Supplemental Figure 7) (uncorrected *p* > .001). However, relative to rest, each task showed a unique pattern of change in tSD, partially mirroring regions of task‐modulation, particularly evident in motor (Figure 5d), and visual regions (Figure 5e,f). Negative task effects in the posterior cingulate cortex and precuneus (Figure 5a–c) paralleled decreases in tSD across all tasks in Figure 5d–f. These regions also had negative tSD values after task effects were regressed (Supplemental Figure 8a–c).

**FIGURE 6:**
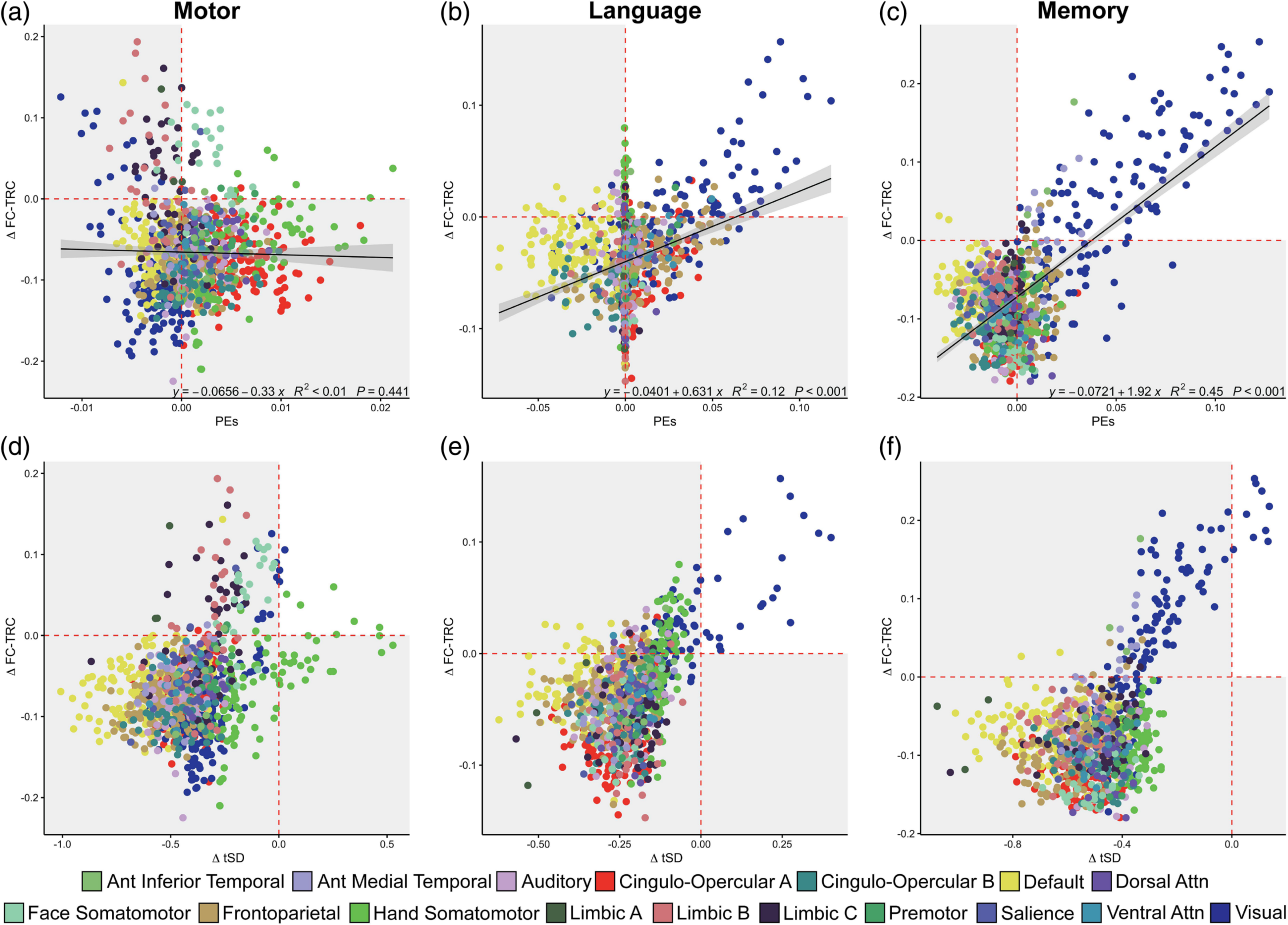
Specific task effects and temporal standard deviation (tSD) associate with relative functional connectivity (FC) reliability. (a–c) Parameter estimates (PEs), from group‐level general linear model (GLM) outputs, did not associate with delta FC‐test–retest correlation (TRC) for the motor task (a), but showed a positive association for the language (b) and memory (c) tasks. (d–f) Relationships between delta FC‐TRC and delta tSD across tasks. There was no discernible association between delta TRC and delta tSD across all parcels, though positive associations are suggested within specific networks, for example, during the motor task (d) in  hand somatomotor network parcels. For the language task (e), visual network regions that showed positive delta tSD also showed positive delta TRC. (f) For the memory task, visual network regions showed increased TRC and larger, though still generally negative changes, in tSD. Overall, DMN regions showed the largest decrease in tSD without a concomitant reduction in TRC.

In Figure 6, we first consider associations between task effects (PEs) and changes in FC‐TRC (top row). PEs, driven by parcels in the visual network, associated positively with ∆ FC‐TRC for both the language (*β* = 6.31e^−1^, *R* ^2^ = .12, uncorrected *p* < .001) and memory tasks (*β* = 1.92, *R* ^2^ = .45, uncorrected *p* < .001). For the motor task, no significant relationship was observed between PEs and ∆ FC‐TRC (*R* ^2^ < .01, uncorrected *p* > .05). Eliminating the low reliability FC networks, that is, limbic A, B, C, we provide corresponding evidence in Supplemental Figure 9, that across networks, associations between both PEs and tSD with FC‐TRC is generally positive across all three tasks. Of note, across the language and memory tasks, a greater positive relationship occurred between PEs and FC‐TRC in Supplemental Figure 9b,c, as compared with the motor task, in Supplemental Figure 9a, showing a more variable trend across networks between task effects and FC‐TRC.

By modulating regional signals, tasks also alter tSD; in the bottom row we consider associations between changes in FC‐TRC and tSD. We showed that for task‐driven parcels, increases in ∆ FC‐TRC occurred in parcels with increases in ∆ tSD, though we note that relative to rest, tSD was generally attenuated, as has previously been noted (Ito et al., 2020). Based on a visual assessment of the data, suggesting a nonlinear relationship, we did not fit linear models to assess the relationship between ∆ FC‐TRC and ∆ tSD. Tasks selectively altered ∆ FC‐TRC and ∆ tSD: regions activated by the demand of a specific task tend to show greater increases in ∆ FC‐TRC and lower attenuation of ∆ tSD, for example, motor regions for the motor task and visual regions for both the language and memory tasks. The relationship between ∆ tSD and ∆ FC‐TRC after task regression is shown in Supplemental Figure 8d–f. After regressing task effects, similar patterns were noted of ∆ FC‐TRC typically increasing with ∆ tSD, but an overall negative shift was found for both measures across all three task states.

### 3.5 Factors contributing to FC reliability across rest and task states

**FIGURE 7:**
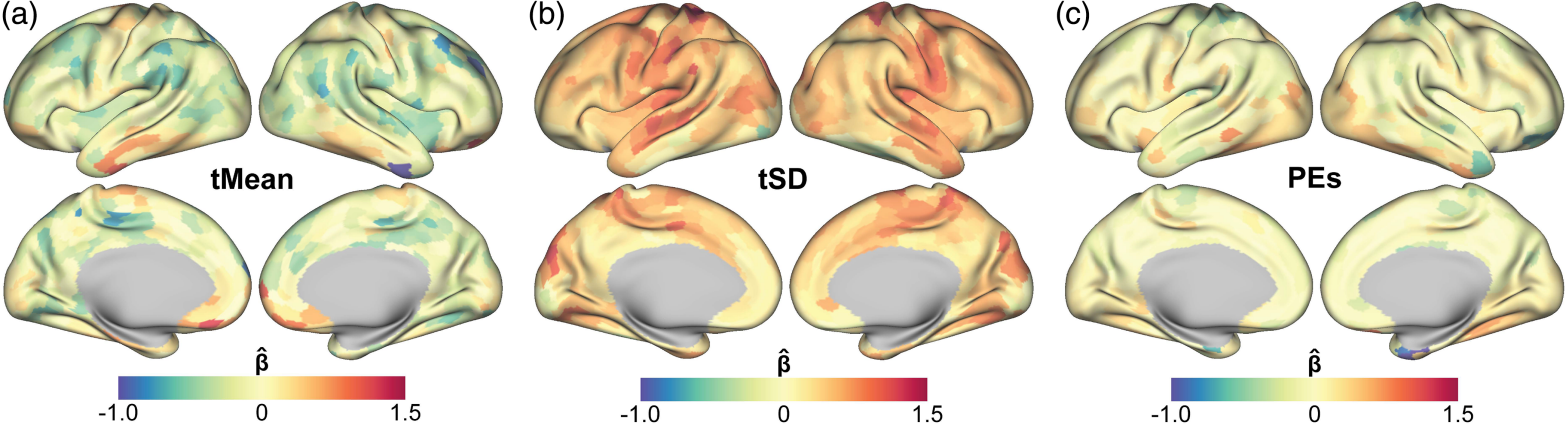
Regional associations with functional connectivity test–retest correlation (FC‐TRC) across tasks for (a) tMean, (b) temporal standard deviation (tSD), and (c) parameter estimates (PEs). A linear mixed effect model was used to determine contributions from tMean, tSD, and PEs to FC‐TRC across all tasks and rest, with standardized predictors. Surface maps show specific positive and negative regional associations for tMean and PEs and relatively broad positive associations for tSD.

Across the brain, FC reliability is associated with tMean, and, to a lesser extent, with tSD and task effects. We next considered the relative association of each of these factors with each region's FC reliability across individuals and tasks, shown in Figure 7. We found that in parcels where signal dropout is known to be large and spatially variable across individuals, that is, ventromedial prefrontal and inferior temporal regions and in motor regions, tMean has a positive association with FC‐TRC. Most other regions showed a negative or negligible association between tMean and FC‐TRC. tSD generally had positive associations with FC‐TRC across the brain. Task PEs had positive associations with FC reliability in specific regions that were engaged differentially across tasks, that is, occipital visual regions.

## 4 DISCUSSION

This study adds to prior work on FC reliability by considering where and how tasks impact regional FC reliability. Overall, our findings suggest that during tasks FC reliability decreases broadly across the brain, except for specific regions that overlap with patterns of task engagement. tSD showed positive associations with FC‐TRC in both across‐brain and region‐wise across‐task analyses. Regional task‐related increases in reliability were effectively removed when task regression was performed. Given challenges in identifying robust FC‐behavior associations (Marek et al., 2022), there is much interest in understanding factors that can help to increase FC reliability. There is great potential in leveraging task fMRI data to increase reliability for FC studies (Elliott et al., 2019; Wang, Ren, et al., 2017; Xu et al., 2016), and it has been noted that tasks tend to increase tolerability and compliance (Shah et al., 2016; Vanderwal et al., 2015; Wang, Han, et al., 2017). Our findings suggest that there is a complex trade‐off when using tasks instead of rest for FC analyses, and that impacts on reliability are task‐ and region‐specific.

Considering the spatial pattern of FC reliability at rest, we generally replicated previous findings (Elliott et al., 2019; Mueller et al., 2015; Noble et al., 2019; Wang, Ren, et al., 2017; Zuo & Xing, 2014), suggesting that FC reliability is greater in higher order cognitive networks and lower in sensory and motor networks. While previous work has linked low tSNR to challenges of identifying robust network parcellations in high dropout regions (Yeo et al., 2011), it has also been observed that tSNR and reliability maps are not identical (Mueller et al., 2015), with some regions showing high tSNR but relatively low reliability. We echo this finding of FC reliability at rest associating nonlinearly with tSNR, on both a parcel‐wise and network‐wise level. Once low reliability networks, limbic A, B, and C are removed from analysis, we observed a negative association between tSNR and FC reliability, driven most prominently by attention and auditory network regions. Delineating the two factors used to compute tSNR—tMean and tSD—we found FC reliability strongly associated with regional signal mean and to a lesser extent with tSD. These relationships hold both overall and across networks after excluding regions in the least reliable networks, where low signal mean is likely related to signal dropout. The components of tSNR make separable influences on FC reliability, contributing to the nonlinear associations observed between reliability and tSNR. This distinction is important because different approaches can be used to target these parameters. Regional signal drop out can be mitigated through strategies such as multi‐echo fMRI (Lynch et al., 2021), while tSD is potentially amenable to manipulation via task.

Comparing vertex‐wise reliability maps between task and rest, reliability generally decreased during tasks. Certain regions, such as face and hand somatomotor regions, showed increased reliability during the motor task, and visual regions showed increased reliability during the language and memory tasks. Follow‐up analyses showed that changes in FC‐TRC across regions was greater for regions engaged by the task, and that this may relate to changes in tSD. However, we note that the relationship between delta FC‐TRC and delta tSD was complex. Given that tSD was attenuated across most parcels, and some regions (e.g., in the DMN) showed proportionally greater attenuation of tSD than FC‐TRC, an overall nonlinear relationship between ∆ tSD and ∆ FC‐TRC was found across the brain. Previous examinations of task impact on signal variability have shown reduced signal variability during tasks relative to rest (Ito et al., 2020) in the context of task‐regressed data, and that task‐regressed data collected during tasks had broadly attenuated FC relative to rest. Our work extends these findings to considerations of reliability, with implications for study design in connectomics.

Prior work has established FC reliability increases with increasing data per participant (Birn et al., 2013; Gordon et al., 2017; Laumann et al., 2015; Noble, Spann, et al., 2017; Xu et al., 2016) and participant alertness (Noble et al., 2019). Studies show that increases in scan length from the typical 5‐min scan to >30 min demonstrate vast improvements in reliability (Birn et al., 2013; Laumann et al., 2015). Aggregating data across rest and task states has also been suggested as an approach to maximize reliability in studies that have collected fMRI under different task conditions (Cho et al., 2021). Previous work has defined personalized functional networks by concatenating across tasks (Cui et al., 2022) since network topography is largely affected by individual factors rather than task states (Gratton et al., 2018). Considering the impact on reliability of specific task concatenation patterns, Cho et al. (2021) found changes in FC reliability based on acquisitions and concatenation techniques. They suggested increases in reliability occur from concatenating shorter scans across similar task conditions rather than across one long scan or combining fMRI data across multiple task conditions. Moreover, they contrasted results with and without GSR, as well as with and without task regression, and found similar trends in reliability across their rest and hybrid task datasets (Cho et al., 2021). Though there are mixed conclusions on the impact of GSR on test–retest reliability (Murphy & Fox, 2017; Shirer et al., 2015), and some studies suggest GSR may decrease FC reliability (Noble et al., 2019, 2021), typically GSR increases the consistency of within‐subject FC across multiple scans (Song et al., 2012) and enhances signal‐noise separation (Shirer et al., 2015). Hence, the use of GSR for this specific study using the MSC dataset is appropriate, as it may aid in removing reliable artifact (Li et al., 2019; Noble et al., 2019). Our findings add to this work by suggesting that regional impacts on reliability are likely going to be affected by the specific set of tasks combined. Moreover, the pattern of reliability across the brain can be considered and potentially mitigated to reduce analytic bias related to varied reliability across the brain (Mueller et al., 2015).

The MSC dataset contains relatively long scan times per participant, known to improve FC‐TRC measures (Gratton et al., 2020; Laumann et al., 2015). In addition, neuroimaging studies increasingly employ multiband (MB) acquisition sequences to improve temporal resolution. Though higher MB factors may worsen SNR (Risk et al., 2021) and alter the reliability of FC measures, prior work has assessed the effect of varying MB factors on FC reliability. The literature suggests a MB acceleration factor of four improves cortical FC reliability, while a single band sequence is preferred for subcortical regions (M. Cahart et al., 2023; M.‐S. Cahart et al., 2022). Further, the combination of multiband and multiecho sequences promise to yield higher reliability estimates than MB sequences alone (Cohen et al., 2021; Fazal et al., 2023; Lynch et al., 2020). Congruent to increasing scan lengths and emerging neuroimaging protocols, our findings postulate that task conditions also increase FC‐TRC in specific regions and the choice of task used in a study may directly impact FC reliability of those regions.

Prior work has shown changes in reliability across known networks for different tasks relative to rest (O'Connor et al., 2017), with increased reliability for the visual network during both naturalistic and visually‐driven (i.e., Inscapes Vanderwal et al., 2015]) conditions and increased reliability for the FPN during both a flanker task and rest. The type and range of tasks available within the MSC dataset are limited; therefore, our study was not able to directly contrast naturalistic viewing tasks with more traditional task designs. Given that naturalistic paradigms contain engaging storylines, they may drive engagement and brain activity more strongly and broadly than both rest and traditional task designs (Finn, 2021; Meer et al., 2020; Sonkusare et al., 2019), thus leading to increases in reliability. Such improvements in reliability were found across all brain regions in a study that compared test–retest reliability of FC measures between movie‐watching and rest (Wang, Ren, et al., 2017). Depending on the study question, naturalistic paradigms may be a worthwhile alternative to rest (Finn, 2021); whereas researchers who intend to target specific brain regions or networks may prefer more traditional task designs targeting a narrower set of brain regions, given the preferential improvements in reliability based on task choice.

Studies that combine task and rest data have often used task regression to reduce task‐evoked effects shared across regions that may confound “intrinsic” FC (Al‐Aidroos et al., 2012; Gratton et al., 2016; Ito et al., 2020). We found that regressing task effects from task‐state time courses dampened FC reliability across the whole brain relative to nonregressed data, and that as expected, task regression eliminated positive task‐related changes in tSD. Overall, this suggests that task regression eliminates any potential reliability gains that occur from using task relative to rest for FC analyses. Our findings are in line with observations in Cho et al. (2021), in which nonregressed data across six task conditions had greater reliability than their regressed counterparts. Task regression may be theoretically important in study design in order to reduce false positive correlations between regions and increase the detection of associations between behavior and intrinsic connectivity (Cole et al., 2019). However, prior work has also shown that compared with nonregressed task data, task‐regressed time courses had worse prediction of individual differences in behavioral outcomes (Zhao et al., 2023), and that this advantage was task‐specific, that is, only task states that associated with a particular behavioral outcome had increased predictive ability. We note that our study focused on the question of FC reliability rather than the impact of using tasks to evoke specific task‐relevant patterns of FC. Even though task regression has been used to recover intrinsic or “background connectivity” during task states, for certain studies (A. S. Greene et al., 2020), it may be necessary to retain task‐related activations in FC to assess the relationship between task‐evoked activations and brain‐phenotypes.

## 5 LIMITATIONS

As noted above, not only does the MSC dataset have a narrow range of tasks, but also the duration of tasks, especially for the motor task (e.g., 7.8 min per day), is limited. Since the motor task was the limiting factor in concatenating scans, this could have caused a loss of temporal signal continuity (Arbabshirani et al., 2019; Kumar et al., 2016), thereby decreasing our potential to reach the temporal upper bounds of FC reliability. It is important to note, after exclusion, our analysis included only nine healthy young adults and thus our findings may not be generalizable. Though our findings may not extend to pediatric or clinical populations, it has been suggested that both developmental populations and populations with neuropsychiatric disorders, exhibit lower overall FC reliability than their adult or neurotypical counterparts (Blautzik et al., 2013; Herting et al., 2018; Somandepalli et al., 2015). Finally, our study was interested in precise surface‐level mapping of task associations; hence, we did not analyze subcortical regions. We focused on mapping reliable differences in cortical regions and note that the tasks used for the MSC study are not specifically designed to engage subcortical structures. Prior work has established that edges belonging to subcortical regions are the least reliable (Noble, Spann, et al., 2017) and future work should consider subcortical structures in detail.

## 6 CONCLUSIONS

To summarize, this study examined how tasks influence fMRI‐FC reliability in a densely sampled dataset. We found that while tasks broadly dampened FC reliability, specific task‐engaged regions tended to show increased reliability and signal variation. Our results can inform study design for maximizing reliability in connectomics research, including widely used practices such as task‐effect regression and across‐task concatenation.

## AUTHOR CONTRIBUTIONS

Shefali Rai: Conceptualization, methodology, validation, formal analysis, investigation, writing ‐ original draft, review & editing, visualization, funding acquisition. Kirk Graff: Investigation, writing ‐ review and editing. Ryann Tansey: Investigation, writing ‐ review and editing. Signe Bray: Conceptualization, methodology, investigation, supervision, project administration, funding acquisition, writing ‐ review and editing.

## FUNDING INFORMATION

This work was supported by an Alberta Graduate Excellence Scholarship and an NSERC‐CREATE Training Scholarship awarded to SR; and an NSERC Discovery Grant to SB.

## CONFLICT OF INTEREST

The authors declare no conflict of interest.

## Supporting information


**SUPPLEMENTAL FIGURE 1.** FC reliability for each of the 9 MSC participants
Variations in test–retest correlation for each MSC participant on a vertex‐wise level. Consistent with Figure 1a, brain regions such as precuneus, superior frontal lobe, and inferior parietal lobe depict greater test–retest correlation as compared to brain regions within the motor cortex and temporal lobe.

**SUPPLEMENTAL FIGURE 2.** Detailed functional network topography
Manually assigned network names, using the original Midnight Scan Club MSCavg network names for reference, corresponding to each of the 17 network outputs from Infomap. Networks for MSCgroup were visualized on the group‐averaged inflated left and right hemisphere surfaces using Connectome Workbench's GUI‐based visualization platform.

**SUPPLEMENTAL FIGURE 3.** Across participant standard deviation of FC reliability
Standard deviation of test–retest correlation shows the opposite effect of test–retest correlation from Figure 1 A, with regions of the motor cortex and temporal lobe illustrating greater variability across individuals.

**SUPPLEMENTAL FIGURE 4.** Variability of FC reliability associations with temporal signal‐to‐noise ratio (tSNR), temporal mean signal (tMean), and temporal standard deviation (tSD) across each network during rest
Linear regression analyses were performed on a network‐wise level, excluding low reliability networks (limbic A, B, and C). Standardized beta estimates are compared across networks for each panel. A) Attention and auditory networks drive the negative relationship between SNR and FC‐TRC during rest. B) Across most networks, tMean associates positively with FC‐TRC. C) The relationship between tSD and FC‐TRC is highly variable across networks, yet overall generally positive.

**SUPPLEMENTAL FIGURE 5.** Regional variation in temporal mean signal (tMean) and temporal standard deviation (tSD) associate with FC‐TRC during tasks
Parcel‐wise associations between FC‐TRC and tMean (A, B, C) and tSD (D, E, F) were fitted using linear regression across all 3 tasks. Analogous to rest, tMean had a significant positive relationship with FC‐TRC across all parcels for all 3 tasks. tSD did not have a significant relationship with FC‐TRC for motor and language tasks **(D, E)**, though had a small but significant relationship with FC‐TRC for the memory task **(F).**

**SUPPLEMENTAL FIGURE 6.** Task‐regression further decreases relative FC reliability
A, B, C) Vertex‐wise relative FC‐TRC calculated using the difference between task‐regressed and rest FC‐TRC. D, E, F) Relative FC‐TRC shown on a parcel‐wise level for each of the 17 networks show dampened values across networks as compared with nonregressed task data. Dotted lines demarcate the zero‐x‐axis line, indicating parcels to the left of this line decrease in FC‐TRC compared to rest, and parcels to the right increase in FC‐TRC relative to rest. Bolded lines represent mean relative FC‐TRC values across networks. Relative to nonregressed data, fewer parcels showed a positive change in reliability with task.

**SUPPLEMENTAL FIGURE 7.** Change in relative temporal mean (tMean) and its associations with FC reliability across tasks
A, B, C) Relative temporal mean signal calculated as the difference between tasks and rest tMean across the cortical surface on a vertex‐wise level. D, E, F) No significant positive relationship was obtained between relative tMean and relative FC‐TRC for all tasks fitted using a linear regression model on a parcel‐wise level. Dotted lines demarcate the zero‐x‐axis and zero‐y‐axis lines.

**SUPPLEMENTAL FIGURE 8.** Relative temporal standard deviation (tSD) and its relationship with FC reliability across task‐regressed data
A, B, C) Relative tSD computed as the difference between all tasks and rest across the cortical surface on a vertex‐wise level. D, E, F) In general, relationships indicated in nonregressed task data were attenuated. Dotted lines demarcate the zero‐x‐axis and zero‐y‐axis lines.

**SUPPLEMENTAL FIGURE 9.** Variability of relative FC reliability associations with task effects (PEs) and relative temporal standard deviation (tSD) across each network during task conditions
Linear regression analyses were performed on a network‐wise level, excluding low reliability networks (limbic A, B, and C). Standardized beta estimates are compared across networks for each panel. A) For the motor task, task effects vary greatly with FC‐TRC across networks, with a slightly positive overall relationship. B, C) Language and memory task effects associate positively with across most networks. D, E, F) Relative tSD relates positively to relative FC reliability across most networks for all three tasks.

**SUPPLEMENTAL TABLE 1.** Censored volumes for each MSC participant across all tasks
**SUPPLEMENTAL TABLE 2**. Task data concatenation across all MSC participants
**SUPPLEMENTAL TABLE 3**. Task accuracy for each MSC participant

## Data Availability

The data that support the findings of this study are openly available in OpenNeuro at https://openneuro.org/datasets/ds000224/versions/1.0.4, reference number ds000224.
