# Supplementary material for: How do tasks impact the reliability of fMRI functional connectivity?
Source: Hum Brain Mapp. 2024 Feb 13;45(3):e26535. doi: 10.1002/hbm.26535 (PMC10884875; doi:10.1002/hbm.26535)
Supplement: Supplementary file 10 — SUPPLEMENTAL TABLE 1. Censored volumes for each MSC participant across all tasks SUPPLEMENTAL TABLE 2. Task data concatenation across all MSC participants SUPPLEMENTAL TABLE 3. Task accuracy for each MSC participant [file HBM-45-e26535-s007.docx]

**Supplemental Tables**

| **Participant** | **Total Volumes > 0.2 mm FD** | **Total % Volumes > 0.2 mm FD** | **Total Volumes across all tasks** | **Missing Data** | **Rest Volumes > 0.2 mm FD** | **Motor Volumes > 0.2 mm FD** | **Language Volumes > 0.2 mm FD** | **Memory Volumes > 0.2 mm FD** |
| --- | --- | --- | --- | --- | --- | --- | --- | --- |
| **MSC01** | 724 | 4.08 | 17730 | N/A | 419 | 75 | 170 | 60 |
| **MSC02** | 189 | 1.15 | 16374 | Motor run 2: ses-func09, Glass run 1: ses-func04, Glass run 2: ses-func03, Glass run 2: ses-func04, Memory faces: ses-func04, Memory words: ses-func04, Memory words: ses-func09, Memory scenes: ses-func04 | 64 | 34 | 56 | 35 |
| **MSC03** | 1396 | 7.87 | 17730 | N/A | 598 | 184 | 322 | 292 |
| **MSC04** | 127 | 0.72 | 17730 | N/A | 27 | 23 | 44 | 33 |
| **MSC05** | 222 | 1.25 | 17730 | N/A | 82 | 41 | 72 | 27 |
| **MSC06** | 238 | 1.34 | 17729 | Rest: ses-func08 is missing 1 volume (817 total) | 36 | 37 | 138 | 27 |
| **MSC07** | 296 | 1.67 | 17730 | N/A | 134 | 97 | 32 | 33 |
| **MSC08** | 2471 | 13.94 | 17730 | N/A | 1809 | 139 | 350 | 173 |
| **MSC09** | 814 | 4.59 | 17730 | N/A | 398 | 204 | 96 | 116 |
| **MSC10** | 1499 | 8.51 | 17609 | Memory scenes: ses-func06 | 934 | 81 | 265 | 219 |

**Supplemental Table 1 – Censored volumes for each MSC participant across all tasks**

Using a framewise displacement (FD) threshold of 0.20 mm, the total number of censored volumes across all tasks and rest for each MSC participant is listed. Missing data information for each task across participants is also shown. MSC08 was excluded from analysis due to ~14% of total volumes contaminated with motion.

|  | **MSC01** | **MSC02** | **MSC03** | **MSC04** | **MSC05** | **MSC06** | **MSC07** | **MSC08** | **MSC09** | **MSC10** |
| --- | --- | --- | --- | --- | --- | --- | --- | --- | --- | --- |
| **Session 1** | 199 | 202 | 196 | 203 | 204 | 206 | 193 |  | 194 | **182** |
| **Session 2** | 206 | 189 | 195 | 201 | 203 | 203 | 186 |  | 175 | **166** |
| **Session 3** | 205 | 201 | 189 | 193 | 198 | 206 | 176 |  | **157** | 190 |
| **Session 4** | 199 | 199 | **173** | 204 | 205 | 206 | 187 |  | 176 | 190 |
| **Session 5** | 200 | 204 | 183 | 205 | 185 | 204 | 184 |  | **156** | 184 |
| **Session 6** | 197 | 205 | 177 | 197 | 174 | 206 | **169** |  | 193 | 202 |
| **Session 7** | 191 | 206 | 170 | 200 | 203 | 202 | 187 |  | **121** | 190 |
| **Session 8** | 203 | 205 | 188 | 203 | 198 | **169** | 188 |  | 190 | 195 |
| **Session 9** | 164 | **102** | 185 | 203 | 197 | 200 | 192 |  | 141 | 203 |
| **Session 10** | 206 | 208 | 206 | 208 | 189 | 206 | **168** |  | 188 | 202 |

**Supplemental Table 2 – Task data concatenation across all MSC participants**

For comparisons between task and rest, the number of available and uncensored volumes was matched for each participant, limited by the motor task. Volumes were matched to the participant with the lowest overall volume for each motor session; lowest values are emphasized in bold.

| **Participant** | **Rest Task Accuracy (%)** | **Motor Task Accuracy (%)** | **Language Task Accuracy (%)** | **Memory Task Accuracy (%)** |
| --- | --- | --- | --- | --- |
| **MSC01** | N/A | N/A | 86.36 | 96.90 |
| **MSC02** | N/A | N/A | 87.99 | 97.35 |
| **MSC03** | N/A | N/A | 87.80 | 97.55 |
| **MSC04** | N/A | N/A | 81.21 | 98.15 |
| **MSC05** | N/A | N/A | 81.29 | 96.44 |
| **MSC06** | N/A | N/A | 86.21 | 97.45 |
| **MSC07** | N/A | N/A | 79.92 | 95.32 |
| **MSC08** | N/A | N/A | 83.48 | 96.76 |
| **MSC09** | N/A | N/A | 87.35 | 97.73 |
| **MSC10** | N/A | N/A | 86.11 | 96.55 |

**Supplemental Table 3 – Task accuracy for each MSC participant**

Percentage of response accuracy for each task across all sessions for all 10 MSC participants, obtained from error response data in the original MSC dataset. High overall accuracy was evident across all participants.

**Supplemental Figure Legends**

**Supplemental Figure 1 – FC reliability for each of the 9 MSC participants**

Variations in test-retest correlation for each MSC participant on a vertex-wise level. Consistent with Figure 1A, brain regions such as precuneus, superior frontal lobe, and inferior parietal lobe depict greater test-retest correlation as compared to brain regions within the motor cortex and temporal lobe.

**Supplemental Figure 2 – Detailed functional network topography**

Manually assigned network names, using the original Midnight Scan Club MSCavg network names for reference, corresponding to each of the 17 network outputs from Infomap. Networks for MSCgroup were visualized on the group-averaged inflated left and right hemisphere surfaces using Connectome Workbench’s GUI-based visualization platform.

**Supplemental Figure 3 – Across participant standard deviation of FC reliability**

Standard deviation of test-retest correlation shows the opposite effect of test-retest correlation from Figure 1 A, with regions of the motor cortex and temporal lobe illustrating greater variability across individuals.

**Supplemental Figure 4 – Variability of FC reliability associations with temporal signal-to-noise ratio (tSNR), temporal mean signal (tMean), and temporal standard deviation (tSD) across each network during rest**

Linear regression analyses were performed on a network-wise level, excluding low reliability networks (limbic A, B, and C). Standardized beta estimates are compared across networks for each panel. **A)** Attention and auditory networks drive the negative relationship between SNR and FC-TRC during rest. **B)** Across most networks, tMean associates positively with FC-TRC. **C)** The relationship between tSD and FC-TRC is highly variable across networks, yet overall generally positive.

**Supplemental Figure 5** – **Regional variation in temporal mean signal (tMean) and temporal standard deviation (tSD) associate with FC-TRC during tasks**

Parcel-wise associations between FC-TRC and tMean **(A, B, C)** and tSD **(D, E, F)** were fitted using linear regression across all 3 tasks. Analogous to rest, tMean had a significant positive relationship with FC-TRC across all parcels for all 3 tasks. tSD did not have a significant relationship with FC-TRC for motor and language tasks **(D, E)**, though had a small but significant relationship with FC-TRC for the memory task **(F).**

**Supplemental Figure 6 – Task-regression further decreases relative FC reliability**

**A, B, C)** Vertex-wise relative FC-TRC calculated using the difference between task-regressed and rest FC-TRC. **D, E, F)** Relative FC-TRC shown on a parcel-wise level for each of the 17 networks show dampened values across networks as compared with non-regressed task data. Dotted lines demarcate the zero-x-axis line, indicating parcels to the left of this line decrease in FC-TRC compared to rest, and parcels to the right increase in FC-TRC relative to rest. Bolded lines represent mean relative FC-TRC values across networks. Relative to non-regressed data, fewer parcels showed a positive change in reliability with task.

**Supplemental Figure 7 – Change in relative temporal mean (tMean) and its associations with FC reliability across tasks**

**A, B, C)** Relative temporal mean signal calculated as the difference between tasks and rest tMean across the cortical surface on a vertex-wise level. **D, E, F)** No significant positive relationship was obtained between relative tMean and relative FC-TRC for all tasks fitted using a linear regression model on a parcel-wise level. Dotted lines demarcate the zero-x-axis and zero-y-axis lines.

**Supplemental Figure 8 – Relative temporal standard deviation (tSD) and its relationship with FC reliability across task-regressed data**

**A, B, C)** Relative tSD computed as the difference between all tasks and rest across the cortical surface on a vertex-wise level. **D, E, F)** In general, relationships indicated in non-regressed task data were attenuated. Dotted lines demarcate the zero-x-axis and zero-y-axis lines.

**Supplemental Figure 9 – Variability of relative FC reliability associations with task effects (PEs) and relative temporal standard deviation (tSD) across each network during task conditions**

Linear regression analyses were performed on a network-wise level, excluding low reliability networks (limbic A, B, and C). Standardized beta estimates are compared across networks for each panel. **A)** For the motor task, task effects vary greatly with FC-TRC across networks, with a slightly positive overall relationship. **B, C)** Language and memory task effects associate positively with across most networks. **D, E, F)** Relative tSD relates positively to relative FC reliability across most networks for all three tasks.
